# Optimal Homotopy Asymptotic Method for Flow and Heat Transfer of a Viscoelastic Fluid in an Axisymmetric Channel with a Porous Wall

**DOI:** 10.1371/journal.pone.0083581

**Published:** 2013-12-23

**Authors:** Fazle Mabood, Waqar A. Khan, Ahmad Izani Ismail

**Affiliations:** 1 School of Mathematical Sciences, Universiti Sains Malaysia, Penang, Malaysia; 2 Department of Engineering Sciences, National University of Sciences and Technology, PN Engineering College, Karachi, Pakistan; Vrije Universiteit, The Netherlands

## Abstract

In this article, an approximate analytical solution of flow and heat transfer for a viscoelastic fluid in an axisymmetric channel with porous wall is presented. The solution is obtained through the use of a powerful method known as Optimal Homotopy Asymptotic Method (OHAM). We obtained the approximate analytical solution for dimensionless velocity and temperature for various parameters. The influence and effect of different parameters on dimensionless velocity, temperature, friction factor, and rate of heat transfer are presented graphically. We also compared our solution with those obtained by other methods and it is found that OHAM solution is better than the other methods considered. This shows that OHAM is reliable for use to solve strongly nonlinear problems in heat transfer phenomena.

## Introduction

In recent years a great deal of focus has been devoted to non-Newtonian fluid flow and heat transfer because of various applications in various fields of science and engineering. In this regard there has been increased interest in flow and heat transfer problem involving viscoelastic fluids. The mathematical modeling of channel flow of viscoelastic fluid and related heat transfer problems (e.g. hot rolling, extrusion of plastic) have been the focus of considerable research works [1]–[9]. Obtaining velocity and temperature distribution from the mathematical model in an efficient and reliable manner is highly important.

The flow through a porous medium has many applications in science and engineering. For instance in ground water hydrology, reservoir engineering, petroleum engineering, chemical engineering, chemical reactors to agriculture irrigation and drainage and the recovery of crude oil from the pores of the reservoir rocks [10]–[12].

In particular, the characteristics of heat transfer in the case of porous medium is of high importance due to its wide range applications in for example, heat exchangers, transport of heated or cooled fluids, micro-electronic cooling, chemical processing equipment, and porous burners, etc. Many researchers have investigated such phenomena. Example include Trimis [13] who has shown that porous media can be very useful within many applications in energy and heat-engineering and Bassam and Abu-Hijleh [14] who have examined heat transfer from a 2D backward facing step with different porous segments and analyzed the effect of these layers on local and overall Nusselt numbers. Nield and Kuznetsov [15] have analyzed the interaction of two porous layers with the same porosity and permeability but different thermal conductivity effects in forced convection and Nemoda *et al.* [16] studied a porous burner and surface burner numerically with different heat conductivity and power of burners. Pilevne and Aydin [17] have investigated forced convection in axisymmetric channel with different porous layers.

Many analytical methods like homotopy perturbation method (HPM) [18]–[19], Adomian decomposition method (ADM) [20]–[21], homotopy analysis method (HAM) [22] have been successfully applied for different heat transfer phenomena.

Optimal homotopy asymptotic method (OHAM) is an approximate (or semi) analytical technique that is straightforward to use and does not require the existence of any small or large parameter.

The basic idea of optimal homotopy asymptotic method was initially introduced by Marinca and Herisanu [23]. OHAM reduces the size of the computational domain and can be applied to wide variety of problems. OHAM is a consistent analytical tool and it has already been successfully applied to a number of nonlinear differential equations arising in science and engineering. OHAM has been used to study steady flow of a fourth-grade fluid flow through a porous medium [23], oscillators with discontinuities and fractional-power restoring force [24], periodic solutions for the motion of a particle on a rotating parabola [25], thin film flow of a fourth-grade fluid [26], nonlinear heat transfer equations [27], and nonlinear problem in elasticity [28].

By means of OHAM, Islam *et al.* [29] investigated Couette and Poiseuille flows of a third grade fluid with heat transfer analysis, Idrees *et al.* [30] analyzed the KDV equation and Mohsen *et al.* [31] studied viscous flow in a semi porous channel with uniform magnetic field. Ghoreishi *et al.* [32] provided a comparative study for nth-order integro-differential equations, Mabood *et al.* [33], [34] investigated heat transfer in hollow sphere and analyzed boundary layer flow, Khan *et al.* [35] studied thin film flow in porous medium. Babaelahi *et al.* [36] examined the viscoelastic boundary layer fluid flow over a stretching sheet.

In this paper, optimal homotopy asymptotic method is utilized in order to study the flow and heat transfer of a viscoelastic fluid in an axisymmetric channel with porous wall and the influence of various parameters on the dimensionless velocity and temperature.

### Problem Formulation

**Figure 1:**
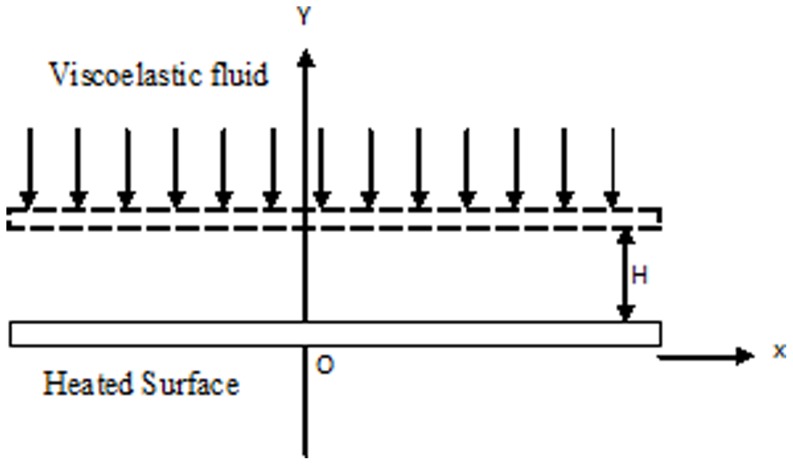
Schematic diagram of the problem.

We consider the phenomena of flow and heat transfer of a viscoelastic fluid in a channel. The schematic diagram is presented in Fig. 1. The x-axis is parallel to the surface of disc and z-axis is normal on it. The porous disc of the channel is placed at Y = +H. The wall that coincides with x-axis is externally heated and other perforated wall viscoelastic fluid is injected consistently in a way to keep cool the heated surface. In this regard, we follow the approach of [9].

We assume that the flow field is stagnation flow with injection. The flow of viscoelastic fluid is considered to be steady, axisymmetric and two-dimensional. The governing equations for the flow and heat transfer are [8]:
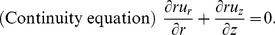
(1)

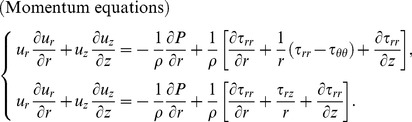
(2)

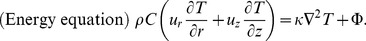
(3)


The boundary conditions for Eqs. (1) – (3) are:
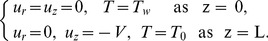
(4)


Where 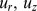 are the velocity components along in 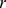 and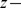 directions, respectively. 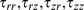 be the components of stress matrix, 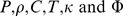 are pressure, density, specific heat, temperature, thermal conductivity and dissipation function respectively, and the dissipation function and stress components are defined as [8]:
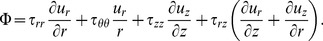
(5)

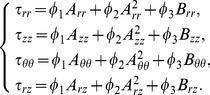
(6)


The similarity solution for Eqs. (1) – (3) with boundary conditions Eq. (4), the dimensionless similarity variables [8]:
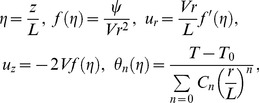
(7)


where prime denotes differentiation with respect to 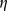.

Using Eq. (7) and eliminating the pressure term, Eqs. (1) – (3) reduced to the following ordinary differential equations or similarity equations.
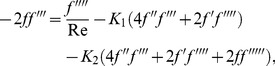
(8)

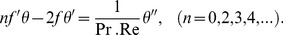
(9)


where 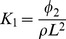,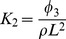 are cross viscosity parameters, Re is the Reynolds number and *Pr* is the Prandtl number. Using Eq. (7) the boundary conditions (Eq. (4)) can be transformed for the considered problem are:
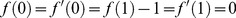
(10)

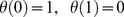
(11)


The above system of Eqs. (8) – (9) with boundary conditions Eqs. (10) – (11) were studied by Kurtcebe and Erim [8] for *K_2_*  =  0. In this paper, we reconsider these equations as:
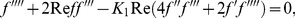
(12)

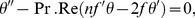
(13)


and solve by means of optimal homotopy asymptotic method.

Quantities of physical interest are the local friction factor, and the local Nusselt number. Physically, 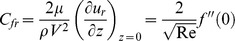 represents the friction factor, and 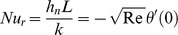 defines the heat transfer rate.

### Basic Principles of OHAM

We review the basic principles of OHAM as expounded in [27]–[28], [34]–[35] and other papers in the following steps:

(i) Let us consider the following differential equation:
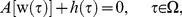
(14)


where 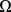 is problem domain, 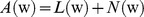, where 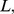 and 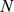 are linear and nonlinear operators respectively, 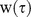 is an unknown function and 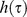
is a known function,

(ii) Construct an optimal homotopy equation as follows:
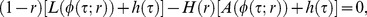
(15)


where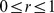 is an embedding parameter,
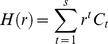
 is an auxiliary function on which the convergence of the solution is dependent. The auxiliary function 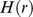 serves also to adjust the convergence domain as well as control the convergence region.

(iii) If 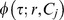 is expanded in a Taylor’s series about 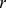, the following an approximate solution is obtained:
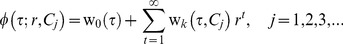
(16)


It has been observed by previous researchers that the convergence of the series Eq. (16) depends upon 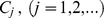. If it is convergent then:
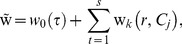
(17)


is obtained.

(iv) Substituting Eq. (17) in Eq. (14), results in the following residual:
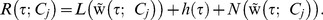
(18)


If 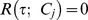, then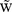 will be the exact solution although for nonlinear problems this will not usually be the case. For determining 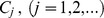 methods such as Galerkin’s method or the method of least squares can be utilized.

(v) Substitution of these constants into Eq. (17) results in the approximate solution.

### Solution of the problem via OHAM

According to the OHAM, Eqs. (12) – (13) can be written as:
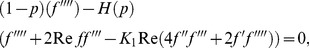
(19)

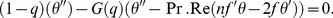
(20)


where primes denote differentiation with respect to 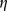.

We consider 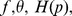 and 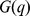 as follows:
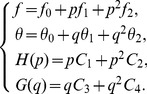
(21)


Using Eq. (21) in Eqs. (19–20) and after simplification as well as rearranging the terms based on the powers of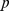 and 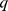, we obtain zeroth, first, and second order problems are:

Zeroth order problem:
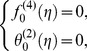
(22)


with boundary conditions:
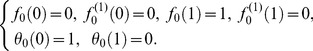
(23)


Its solution is: 
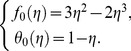
(24)


First order problem:
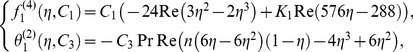
(25)


with boundary conditions:
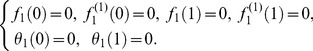
(26)


Its solution is: 
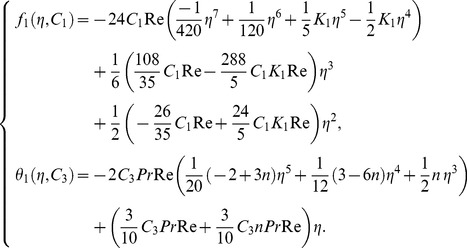
(27)


and so on …

Set 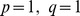, to obtain the three terms solution using OHAM for dimensionless velocity and temperature profile as:
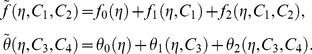
(28)


The method of least squares is used to obtain the convergence-control parameters 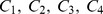 in Eq. (28), i.e.
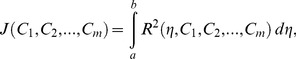
(29)


where 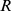 is the residual,
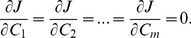
(30)


In case of 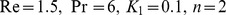, then the values of 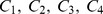 are given below: 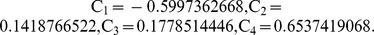


## Results and Discussion

In this paper, we have successfully employed OHAM to obtain the approximate analytical solutions of flow and heat transfer problem for viscoelastic fluid in a channel with porous wall. Various values of different controlling parameters for both dimensionless velocity and temperature profiles are obtained. The approximate analytical solution for dimensionless velocity and temperature using OHAM are compared with those obtained by HAM [9] and numerical shooting method [9] shown in Tables 1 and 2. These results indicate that OHAM is a feasible and efficient technique for problems arising in heat transfer although more studies need to be conducted. Percentage of error is calculated for obtained OHAM solutions in Tables 1–2. The percentages reflect that our solution is more accurate than HAM [9] solution.

**Table 1:** Comparison of 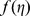 values using different methods.

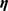	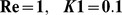				
	OHAM (Present results)	NM [9]	HAM [9]	% error HAM	% error OHAM
0.0	0	0	0	0	0
0.1	0.3021760	0.3021792	0.03021752	0.00132371	0.00105897
0.2	0.11348615	0.11348995	0.11348557	0.00385937	0.00334831
0.3	0.23672114	0.23672600	0.23671190	0.00595625	0.00205300
0.4	0.38448353	0.38449935	0.38448227	0.00444214	0.00411444
0.5	0.54036989	0.54036906	0.54037008	0.00018876	0.00015359
0.6	0.68889001	0.68889398	0.68891470	0.00203772	0.00057628
0.7	0.81730234	0.81729920	0.81731584	0.00203597	0.00038419
0.8	0.91604548	0.91604363	0.91604691	0.00035806	0.00020195
0.9	0.97845326	0.97845350	0.97845308	0.00004292	0.00001242
1.0	1	1	1	0	0

**Table 2:** Comparison of 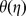
values using different methods.

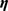	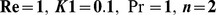				
	OHAM (Present results)	NM [9]	HAM [9]	% error HAM	% error OHAM
0.0	1	1	1	0	0
0.1	0.82706311	0.82706323	0.82706690	0.0004437	0.0000145
0.2	0.66453625	0.66453256	0.66453855	0.0009013	0.0005552
0.3	0.51925531	0.51925353	0.51925913	0.0010784	0.0003428
0.4	0.39448602	0.39448392	0.39448742	0.0008872	0.0005323
0.5	0.29052119	0.29051950	0.29052246	0.0010188	0.0005817
0.6	0.20561711	0.20561368	0.20561841	0.0023004	0.0016681
0.7	0.13697446	0.13697040	0.13697661	0.0045338	0.0029641
0.8	0.08156221	0.08156009	0.08156599	0.0072339	0.0025993
0.9	0.03663756	0.03663979	0.03664378	0.0108898	0.0060862
1.0	0	0	0	0	0

**Figure 2:**
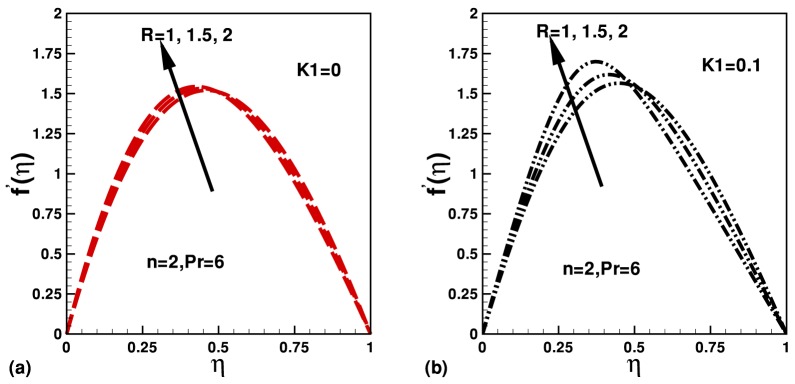
Effects of
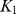
on dimensionless velocity profile for different values of Reynolds number.

**Figure 3:**
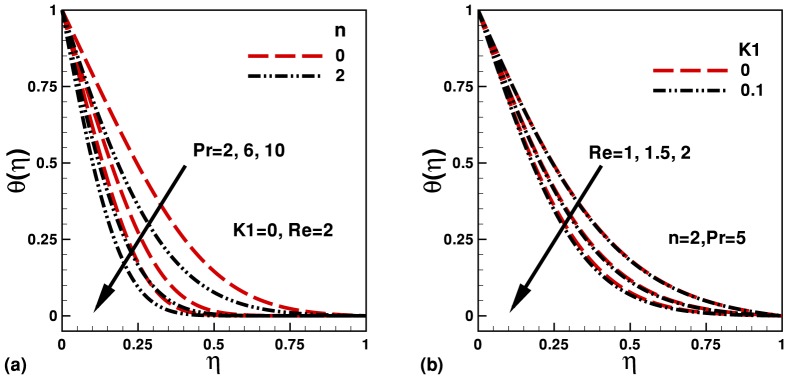
Variation of dimensionless temperature with transverse distance for different values of parameters.

**Figure 4:**
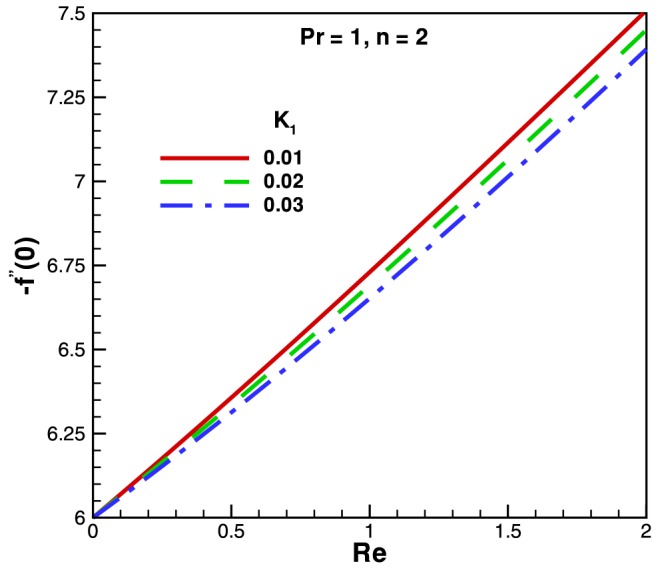
Variation of skin friction coefficient with Reynolds number and different value of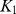.

**Figure 5:**
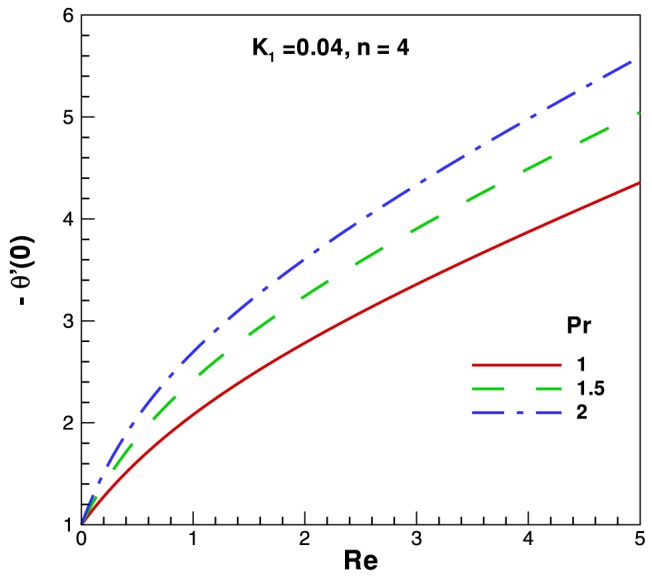
Variation of heat transfer rates with Reynolds number and different values of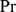.

**Figure 6:**
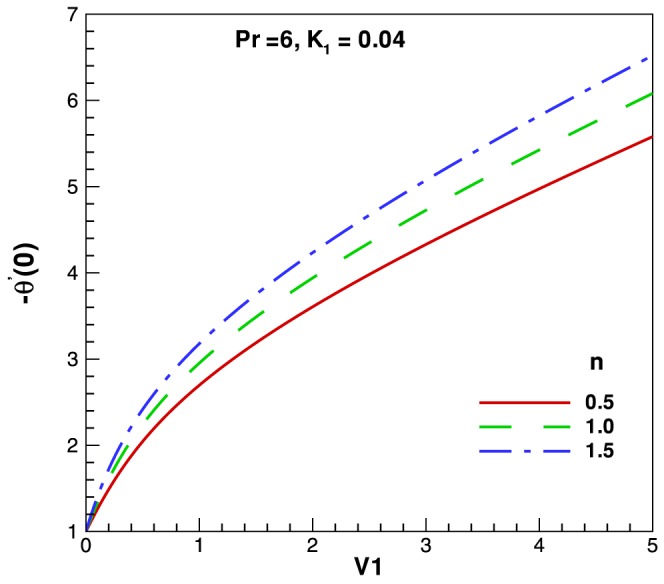
Variation of heat transfer rates with Reynolds number and different values of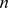.

**Figure 7:**
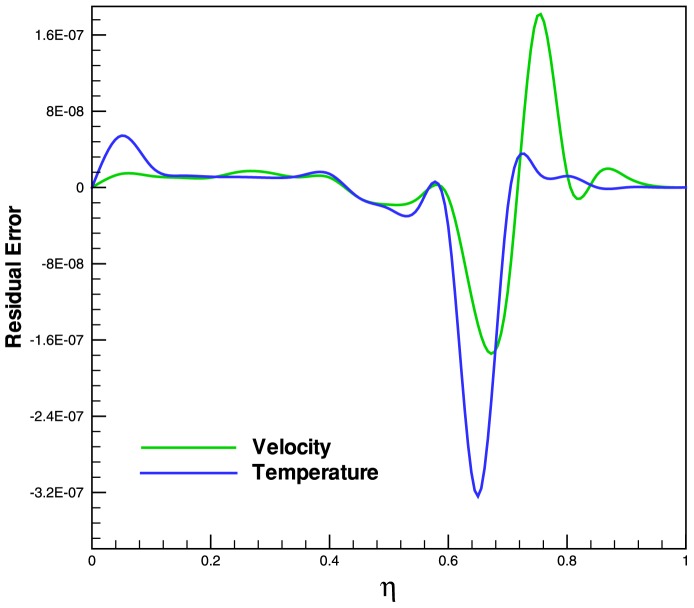
Residual Error of dimensionless velocity and temperature profiles at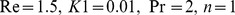.

The influence and effect of different parameters on velocity and temperature are displayed in Figs. 2 and 3. In Figs 2(a) and (b), the effect of parameters 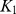 and 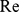 on the dimensionless velocity are displayed while keeping fixed values of parameters 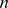 and 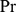. It is observed that the dimensionless velocity at the center of the channel increases with an increase in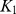. The dimensionless velocity also increases with Reynolds number inside the channel. In Fig. 3(a), the effects of 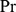
and 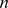 on the dimensionless temperature are displayed for different values of 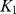 and 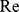. It is found that the dimensionless temperature decreases inside the thermal boundary layer with increasing 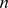 and 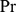. The effects of 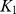 on the dimensionless temperature are found to be negligible whereas the dimensionless temperature decreases with an increase in the Reynolds number. In Fig. 4 we present and highlight the influence of Reynolds and cross viscosity parameter on skin-friction factor. It is notice that skin-friction factor coefficient increases linearly with an increase in 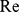 and the opposite nature of skin-friction factor can be observed with the increases in cross viscosity parameter 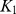. The variation of the Nusselt number (representing the dimensionless heat transfer rate at the surface) is presented for different parameters in Figs. 5–6. Fig. 5 depicts the behavior of heat transfer rates against Reynolds and Prandtl numbers. A monotonic increase in heat transfer rate is observed for increasing values in both parameters. Fig. 6 shows the heat transfer rate against Reynolds and for different fluids according to power law index 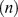. The heat transfer rate increases with the increase in power law index, which indicate that the non-isothermal surface generate higher heat transfer rates. Finally, Fig. 7 shows the residual error for both dimensionless velocity and temperature profiles.

## Conclusion

In this paper, OHAM is successfully employed in order to obtain an approximate solution for flow and heat transfer of viscoelastic fluid in a channel. Effects of different controlling parameters on dimensionless velocity, temperature, skin-friction as well as Nusselt number are investigated. The present OHAM results are compared with existing numerical and HAM result and a good agreement is observed with both methods. These comparisons show that OHAM is an effective technique for the solution of nonlinear problems in heat transfer. We can draw the following conclusion:

- The dimensionless temperature directly proportional to Reynolds number
- The skin-friction factor increases monotonically with Reynolds number
- Rate of heat transfer increases with increase in Prandtl number
